# Errors in Estimating Lower-Limb Joint Angles and Moments during Walking Based on Pelvic Accelerations: Influence of Virtual Inertial Measurement Unit’s Frontal Plane Misalignment

**DOI:** 10.3390/s24165096

**Published:** 2024-08-06

**Authors:** Takuma Inai, Yoshiyuki Kobayashi, Motoki Sudo, Yukari Yamashiro, Tomoya Ueda

**Affiliations:** 1 Health and Medical Research Institute, National Institute of Advanced Industrial Science and Technology, 2217-14 Hayashi-cho, Takamatsu 761-0395, Kagawa, Japan; 2 Human Augmentation Research Center, National Institute of Advanced Industrial Science and Technology, 6-2-3 Kashiwanoha, Kashiwa 277-0882, Chiba, Japan; kobayashi-yoshiyuki@aist.go.jp; 3 Tokyo Research Laboratories, Kao Corporation, 2-1-3 Bunka, Sumida-ku 131-8501, Tokyo, Japan; sudou.motoki@kao.com (M.S.); yamashiro.yukari@kao.com (Y.Y.); ueda.tomoya@kao.com (T.U.)

**Keywords:** gait, joint angle, joint moment, acceleration, misalignment

## Abstract

The accurate estimation of lower-limb joint angles and moments is crucial for assessing the progression of orthopedic diseases, with continuous monitoring during daily walking being essential. An inertial measurement unit (IMU) attached to the lower back has been used for this purpose, but the effect of IMU misalignment in the frontal plane on estimation accuracy remains unclear. This study investigated the impact of virtual IMU misalignment in the frontal plane on estimation errors of lower-limb joint angles and moments during walking. Motion capture data were recorded from 278 healthy adults walking at a comfortable speed. An estimation model was developed using principal component analysis and linear regression, with pelvic accelerations as independent variables and lower-limb joint angles and moments as dependent variables. Virtual IMU misalignments of −20°, −10°, 0°, 10°, and 20° in the frontal plane (five conditions) were simulated. The joint angles and moments were estimated and compared across these conditions. The results indicated that increasing virtual IMU misalignment in the frontal plane led to greater errors in the estimation of pelvis and hip angles, particularly in the frontal plane. For misalignments of ±20°, the errors in pelvis and hip angles were significantly amplified compared to well-aligned conditions. These findings underscore the importance of accounting for IMU misalignment when estimating these variables.

## 1. Introduction

Lower-limb joint angles and moments observed during walking are crucial variables in gait analysis and serve as important indicators for evaluating the risk of orthopedic diseases such as hip and knee osteoarthritis [1,2,3,4,5,6,7]. For example, a higher hip flexion angle during the early stance phase and a lower hip extension angle during the late stance phase have been identified as risk factors for the progression of hip osteoarthritis [7]. Additionally, increased external knee adduction moments have been associated with a higher risk of knee osteoarthritis progression [1]. Therefore, quantifying lower-limb joint angles and moments during daily walking is valuable for the ongoing assessment of orthopedic disease risk.

Typically, joint angles and moments during walking are determined using motion capture systems equipped with multiple cameras and/or force plates [8,9]. However, installing cameras and force plates throughout outdoor environments to continuously measure these variables is impractical. Consequently, a more feasible method is needed for the continuous measurement of lower-limb joint angles and moments in everyday settings.

An alternative approach involves using wearable sensors that measure acceleration and/or angular velocity, such as inertial measurement units (IMUs). These sensors are lightweight and compact, making them convenient for use in various environments. Some researchers have reported promising methods for estimating lower-limb joint angles and moments during walking by attaching IMUs to the lower backs of subjects [10,11].

However, IMUs have the potential disadvantage of misalignment on the body. The misalignment of the IMU on the pelvis in the frontal plane is common and may result from attachment errors during self-placement [12] or from the influence of daily movements [13]. When misalignment occurs due to self-placement errors, a calibration method can be employed during IMU attachment. Conversely, when misalignment is caused by daily movements, applying a calibration method is challenging because the error occurs during these movements (e.g., due to clothing misalignment or external forces).

Previous studies have used three-axis acceleration data from the lower back to estimate lower-limb joint angles and moments during walking [10,11]. Since the misalignment of an IMU in the frontal plane affects acceleration waveforms during walking, it can introduce errors in the estimation of lower-limb joint angles and moments. Significant estimation errors may lead to inaccurate evaluations of medical risks, such as the progression of knee and hip osteoarthritis. Therefore, this study aimed to examine the impact of misalignment of a virtual IMU angle with respect to the pelvic frontal plane on estimation errors of lower-limb joint angles and moments during walking. We utilized the AIST Gait Database, which includes data from a motion capture system and force plates but does not use IMUs. This allowed us to precisely simulate the misalignment of the virtual IMU (e.g., 10 degrees) using reflective markers. Thus, we used this database to better understand the phenomenon and its impacts.

## 2. Materials and Methods

### 2.1. Participants

This study included 278 healthy adults (130 males; average age: 51.1 ± 18.1 years; height: 1.63 ± 0.01 m; body mass: 59.4 ± 10.3 kg). The inclusion criteria were as follows: (1) the participant must be able to walk independently, without the use of a walking aid; (2) the participant must have no orthopedic or neurological diseases; (3) the participant must not experience pain in the lower limbs; (4) the participant must have normal or corrected-to-normal vision; and (5) the participant must be over 20 years of age. The experimental protocol was approved by the ethics committee of the National Institute of Advanced Industrial Science and Technology (IRB number: 71120030-E−20150303-002). All participants provided written informed consent before taking part in the experiment.

### 2.2. Experiment

A motion capture system (Vicon, Oxford, UK) with a sampling frequency of 200 Hz, in conjunction with six force plates for ground reaction force (GRF) measurement (AMTI, Watertown, MA, USA) with a sampling frequency of 1000 Hz, was used for this study. The experimenters, who were palpation experts, such as physical therapists, attached 55 reflective markers to each participant according to the protocol described in a previous study [14]. Although the previous study used 57 markers, 2 markers were not applied to the halluces in this study. The participants then walked barefoot along a straight 10 m path in the laboratory at a self-selected speed. Five successful trials were recorded for each participant.

### 2.3. Data Analysis

Raw trajectory data for each marker were exported from Visual 3D software Professional v2023.06.2 (HAS-Motion Inc., Ontario, Canada) and filtered using a fourth-order Butterworth filter with zero lag and a cutoff frequency of 10 Hz [15,16,17,18]. The Butterworth filter for the raw data of the GRFs also had zero lag but used a cutoff frequency of 6 Hz. A gait cycle was defined as the period from one heel contact to the next ipsilateral heel contact, with heel contact timing determined using the vertical GRF.

Local coordinate systems were established for the pelvis [19], thighs [19], shanks [19], and feet [18] using methods described in previous studies. We calculated lower-limb joint angles (Cardan angles [sequence: x-y-z]) using these local coordinate systems (i.e., pelvis angle, and hip, knee, and ankle joint angles for each axis). Additionally, we calculated lower-limb joint moments using inverse dynamics calculations (i.e., hip, knee, and ankle joint moments for each axis).

**Figure 1:**
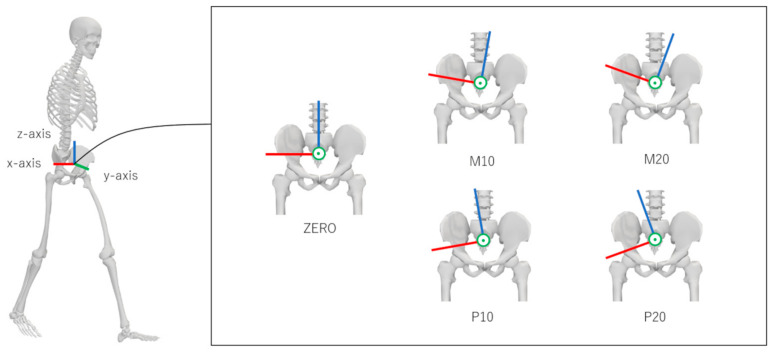
Five angular misalignment conditions considered for the virtual IMU: (1) −20° (M20), (2) −10° (M10), (3) 0° (ZERO), (4) +10° (P10), and (5) +20° (P20). This figure was created in OpenSim 4.4 [20,21].

Three-axis accelerations of a virtual midpoint of the left and right anterior superior iliac spines (MASIS) marker in the global coordinate system were calculated using the filtered marker trajectories (central differential method). Subsequently, the acceleration data in the global coordinate system were transformed into the local coordinate system of a virtual IMU. As shown in Figure 1, five misalignment conditions were set for the virtual IMU: (1) −20° (M20), (2) −10° (M10), (3) 0° (ZERO), (4) +10° (P10), and (5) +20° (P20). In other words, the local coordinate system of the virtual IMU in the ZERO condition was aligned with the local coordinate system of the pelvis. Lower-limb joint angles, moments, and accelerations were time-normalized to 101 points.

### 2.4. Estimation Method for Joint Angles and Moments

**Figure 2:**
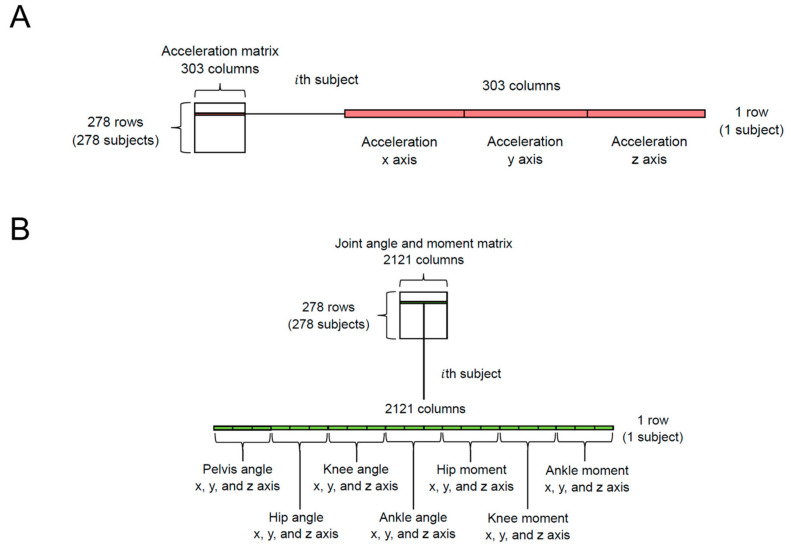
Structures of the (**A**) acceleration matrix and (**B**) joint angle and moment matrix. We used the acceleration matrix as the input and the joint angle and moment matrix as the output.

**Figure 3:**
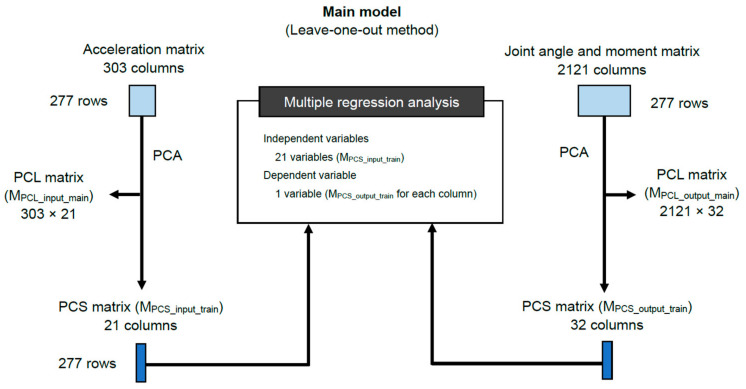
Flowchart of the algorithm to derive the joint angle and moment estimation model. Principal component analysis (PCA) was performed to compress the dimensions of the acceleration, and joint angle and moment matrices. PCL and PCS refer to the principal component loading and principal component score, respectively.

As illustrated in Figure 2, we first prepared the “acceleration matrix” (278 rows × 303 columns) and the “joint angle and moment matrix” (278 rows × 2121 columns). The acceleration matrix contains 303 columns of acceleration data in the x (101 columns), y (101 columns), and z (101 columns) axes. The joint angle and moment matrix comprises 2121 columns, including angles (pelvis, hip, knee, and ankle; 1212 columns = 101 points × 3 axes × 4 segments/joints) and joint moments (hip, knee, and ankle; 909 columns = 101 points × 3 axes × 3 joints). The leave-one-out cross-validation method was applied, resulting in 277 rows in each matrix (Figure 3). Figure 3 illustrates the flowchart of the algorithm used to establish the relationship between the two matrices. Principal component analysis (PCA) was first applied to reduce the dimensions of both matrices. The resulting principal component scores (PCSs) were reduced to dimensions of 21 for the acceleration matrix and 32 for the joint angle and moment matrix. A cumulative contribution rate (CCR) of 80% or 90% is commonly used in PCA to select principal components [22]. In this study, we applied a 90% CCR. Specifically, the CCR for the acceleration matrix was 90.3%, and for the joint angle and moment matrix, it was 90.2%. The 21 PCSs from the acceleration matrix were used as independent variables, whereas 1 of the 32 PCSs from the joint angle and moment matrix served as the dependent variable in linear regression analysis. Thus, 32 linear regression equations were formulated.

For each excluded participant, we calculated 32 PCSs using the participant’s acceleration data, the principal component loading (PCL) matrix (i.e., M_PCL_input_main_; Figure 3), and the linear regression equations. Subsequently, joint angles and moments were reconstructed and estimated using the calculated 32 PCSs of the excluded participant and the PCL matrix (i.e., M_PCL_output_main_; Figure 3). The errors between the estimated and actual (motion capture) joint angles and moments were calculated using the leave-one-out method. The accuracy of the estimated lower-limb joint angles and moments was evaluated using the normalized root-mean-square error ($\bar{NRMSE}$) (%) based on previous studies [23,24,25,26] (normalization was performed using the difference between maximum and minimum values). Additionally, we calculated the ratio of the “corresponding $\bar{NRMSE}$” to the “$\bar{NRMSE}$ in the ZERO condition”.

## 3. Results

Table 1 and Table 2 list the $\bar{NRMSE}$ values and the $\bar{NRMSE}$ ratios for each estimated joint angle and moment. The $\bar{NRMSE}$ ratios for the pelvis and hip angles in the frontal plane under the M20 and P20 conditions were notably higher compared to other variables in the same conditions (22.0 and 13.2, respectively). In contrast, the effects of misalignment on the $\bar{NRMSE}$ ratios for lower-limb joint angles (except for the pelvis and hip joint angles in the frontal plane) and moments were small. The $\bar{NRMSE}$ values for lower-limb joint angles and moments (except for the knee joint moment) in the sagittal plane under the ZERO condition were lower than those in the frontal and horizontal planes under the ZERO condition.

**Figure 4:**
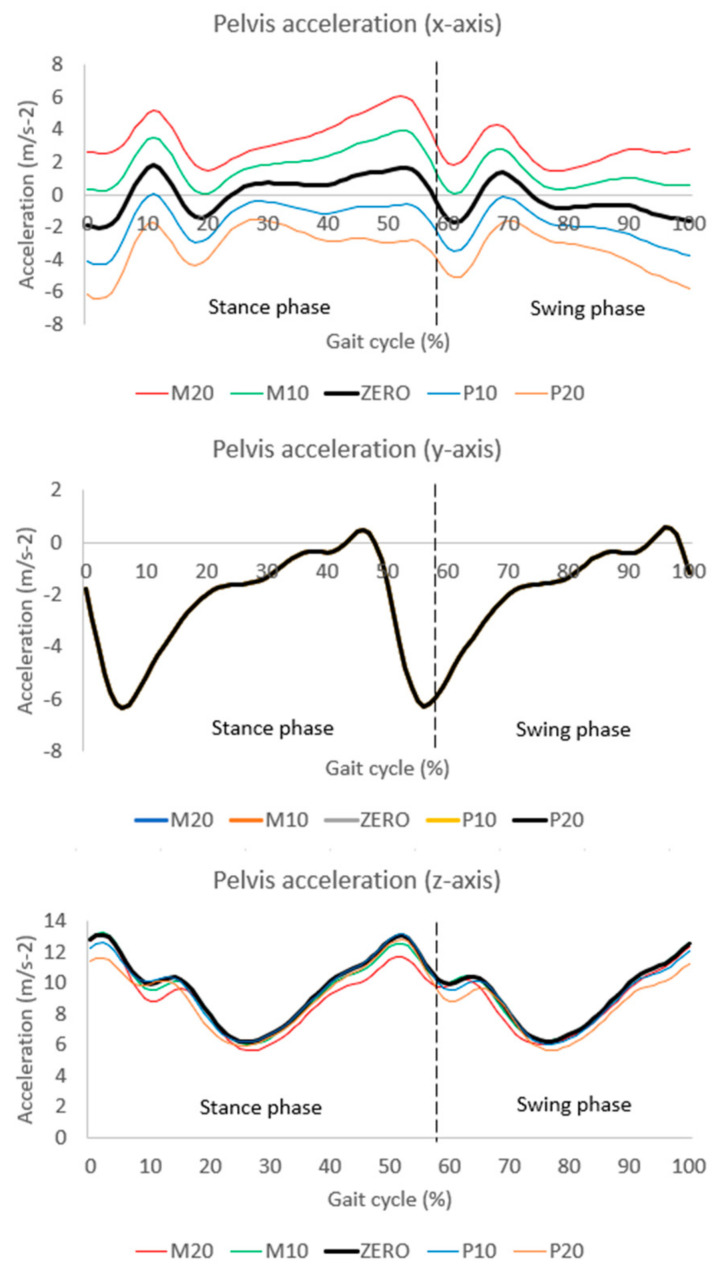
Waveforms of pelvic accelerations during gait: medio-lateral (x-axis), anterior/posterior (y-axis), and superior/inferior (z-axis).

Figure 4 displays the pelvic accelerations for each condition. The accelerations along the y-axis were consistent across all conditions. However, accelerations along the x- and z-axes varied between conditions.

## 4. Discussion

This study used motion capture data to evaluate errors in estimating lower-limb joint angles and moments during walking, specifically focusing on the impact of misalignment of a virtual IMU in the frontal plane on the pelvis. Our results show that an increased angular misalignment of the virtual IMU in the frontal plane significantly affects the estimation errors of pelvis and hip angles. In contrast, the effects of misalignment on the ratio of estimation errors for other joint angles (excluding the pelvis and hip angles in the frontal plane) and moments were minimal. Despite the frequent occurrence of IMU misalignment in the frontal plane, no prior research, to our knowledge, has investigated its effects on the estimation errors of joint angles and moments during walking. Therefore, we believe that our findings are both novel and valuable.

Additional analysis was conducted to elucidate the mechanism behind these findings (see Supplementary Materials). We performed PCAs on the joint angle matrix (including only the pelvis and hip joint angles in the frontal plane) and the acceleration matrix. Subsequently, a multiple regression analysis was performed, with all PCSs from the acceleration matrix as independent variables and PCS1 from the joint angle matrix as the dependent variable. The results revealed a strong relationship between PCV7 of the acceleration matrix and PCV1 of the joint angle matrix. Additionally, PCV7 of the acceleration matrix showed a moderate relationship with acceleration along the x-axis, as indicated by the principal component loadings (Figure S1). Moreover, changes in the misalignment of the virtual IMU in the frontal plane affected acceleration along the x-axis (Figure 4). Consequently, we conclude that variations in pelvis acceleration along the x-axis influenced the estimation errors of pelvis and hip angles in the frontal plane.

Additionally, under the ZERO condition, the $\bar{NRMSE}$ values of lower-limb joint angles and moments (excluding the knee joint moment) in the sagittal plane were smaller than those in the frontal and horizontal planes. This relatively low $\bar{NRMSE}$ values can be explained by two factors: (1) the stronger relationship between walking speed and kinematics (and kinetics) in the sagittal plane [8], and (2) the strong relationship between walking speed and certain principal component values (PCVs) of the acceleration matrix (refer to the Supplementary Materials for details [Additional analysis S2]). Thus, the estimation accuracy of lower-limb joint angles and moments in the sagittal plane is likely higher than in other planes.

However, there are some limitations to the present study. First, previous research has shown that kinematics and kinetics differ between healthy adults and patients with knee or hip osteoarthritis [27,28,29,30,31,32]. This study included only healthy adults, so future research should focus on patients with orthopedic diseases. Second, the study did not use actual IMUs due to challenges in consistently attaching an IMU at appropriate angles across various conditions. Instead, we configured the misalignment of a virtual IMU using data from the motion capture system. While this approach enhances our understanding of the effects of misalignment, evaluating the accuracy of our algorithm with actual IMUs remains important for future research. Finally, we employed only one method (PCA combined with a multiple regression model) to estimate joint angles and moments. Therefore, the main findings of this study may not be generalized to other estimation methods and future research should explore alternative approaches.

## 5. Conclusions

In conclusion, using motion capture data from healthy subjects, we found that increasing the angular misalignment of a virtual IMU on the pelvis in the frontal plane led to significant errors in the estimation of pelvis and hip angles in the frontal plane during walking. Additionally, the effects of misalignment on estimation errors for lower-limb joint angles (excluding pelvis and hip joint angles in the frontal plane) and moments were minimal. These results enhance our understanding of the relationship between IMU misalignment in the frontal plane and errors in estimating joint angles and moments during gait.

## Figures and Tables

**Table 1:** Normalized root-mean-squared-errors (NRMSEs) and ratio of NRMSEs for each joint angle.

	Condition									
	M20		M10		ZERO		P10		P20	
	Mean	(SD)	Mean	(SD)	Mean	(SD)	Mean	(SD)	Mean	(SD)
**NRMSE (Unit: %)**										
**Pelvis**										
Sagittal angle	70.4	(24.5)	39.1	(14.3)	28.6	(8.4)	35.7	(10.8)	56.1	(18.0)
Frontal angle	175.2	(58.3)	89.2	(30.0)	9.3	(5.0)	89.3	(30.2)	175.1	(58.6)
Horizontal angle	37.9	(18.8)	25.2	(14.6)	20.8	(13.6)	28.8	(20.7)	44.6	(28.9)
**Hip**										
Sagittal angle	6.4	(2.9)	5.6	(2.6)	5.1	(2.3)	6.0	(2.6)	9.2	(3.4)
Frontal angle	170.4	(41.7)	87.1	(24.5)	16.2	(8.6)	87.4	(25.7)	171.5	(43.0)
Horizontal angle	89.3	(37.8)	57.1	(30.0)	40.3	(26.3)	58.2	(33.3)	94.9	(42.6)
**Knee**										
Sagittal angle	10.3	(3.4)	7.5	(3.1)	6.1	(2.6)	7.6	(3.0)	11.5	(3.5)
Frontal angle	97.4	(50.1)	63.7	(36.9)	46.2	(26.9)	62.0	(33.0)	98.8	(44.7)
Horizontal angle	86.5	(50.2)	56.3	(38.5)	43.8	(28.4)	53.4	(32.7)	76.3	(42.1)
**Ankle**										
Sagittal angle	13.6	(6.1)	11.6	(5.6)	11.1	(5.5)	12.3	(6.1)	15.3	(7.3)
Frontal angle	51.8	(30.1)	37.2	(23.0)	30.5	(16.7)	35.3	(16.3)	48.2	(19.9)
Horizontal angle	73.4	(42.6)	48.1	(33.3)	37.4	(30.3)	48.4	(40.9)	74.6	(52.2)
**Ratio of NRMSE**										
**Pelvis**										
Sagittal angle	2.6	(0.9)	1.4	(0.4)	1.0	(0.0)	1.3	(0.3)	2.1	(0.7)
Frontal angle	22.0	(9.7)	11.2	(4.9)	1.0	(0.0)	11.2	(5.0)	22.0	(9.8)
Horizontal angle	2.2	(1.3)	1.4	(0.7)	1.0	(0.0)	1.5	(0.7)	2.5	(1.4)
**Hip**										
Sagittal angle	1.4	(0.5)	1.1	(0.3)	1.0	(0.0)	1.3	(0.5)	2.1	(1.1)
Frontal angle	13.2	(7.1)	6.8	(3.7)	1.0	(0.0)	6.7	(3.6)	13.2	(7.0)
Horizontal angle	2.8	(1.4)	1.7	(0.8)	1.0	(0.0)	1.7	(0.8)	2.9	(1.5)
**Knee**										
Sagittal angle	1.9	(0.8)	1.3	(0.4)	1.0	(0.0)	1.3	(0.5)	2.2	(0.9)
Frontal angle	2.5	(1.4)	1.5	(0.8)	1.0	(0.0)	1.6	(0.8)	2.7	(1.5)
Horizontal angle	2.7	(1.9)	1.6	(1.0)	1.0	(0.0)	1.5	(0.9)	2.4	(1.7)
**Ankle**										
Sagittal angle	1.3	(0.5)	1.1	(0.3)	1.0	(0.0)	1.2	(0.3)	1.5	(0.6)
Frontal angle	1.9	(1.0)	1.3	(0.5)	1.0	(0.0)	1.3	(0.5)	1.9	(0.9)
Horizontal angle	3.2	(2.5)	1.8	(1.3)	1.0	(0.0)	1.8	(1.3)	3.1	(2.5)

Note: The ratio of the NRMSE indicates the ratio of the corresponding NRMSE to that in the ZERO condition.

**Table 2:** Normalized root-mean-squared-errors (NRMSEs) and ratio of NRMSEs for each joint moment.

	Condition									
	M20		M10		ZERO		P10		P20	
	Mean	(SD)	Mean	(SD)	Mean	(SD)	Mean	(SD)	Mean	(SD)
**NRMSE (Unit: %)**										
**Hip**										
Sagittal moment	9.1	(2.5)	6.8	(2.4)	6.0	(2.5)	7.3	(3.2)	10.2	(4.1)
Frontal moment	20.3	(5.5)	11.7	(4.7)	8.5	(4.4)	13.0	(7.8)	20.9	(10.2)
Horizontal moment	36.3	(13.4)	19.9	(7.7)	8.4	(3.8)	20.1	(7.5)	37.2	(13.4)
**Knee**										
Sagittal moment	15.5	(8.8)	11.3	(7.0)	9.2	(5.4)	10.6	(5.0)	14.6	(5.3)
Frontal moment	22.0	(13.8)	16.3	(11.2)	13.0	(8.2)	14.4	(6.5)	20.2	(6.3)
Horizontal moment	11.3	(4.8)	9.1	(4.6)	8.0	(4.8)	9.6	(5.8)	13.6	(7.3)
**Ankle**										
Sagittal moment	5.5	(2.4)	5.1	(2.2)	4.8	(2.2)	5.1	(2.2)	6.2	(2.3)
Frontal moment	40.4	(13.8)	28.1	(10.7)	25.0	(16.7)	33.5	(27.0)	49.1	(35.3)
Horizontal moment	14.9	(8.8)	12.3	(8.2)	11.2	(7.4)	12.1	(7.0)	14.7	(6.9)
**Ratio of NRMSE**										
**Hip**										
Sagittal moment	1.7	(0.6)	1.2	(0.3)	1.0	(0.0)	1.3	(0.3)	1.8	(0.7)
Frontal moment	3.0	(1.5)	1.7	(0.9)	1.0	(0.0)	1.7	(0.9)	2.9	(1.5)
Horizontal moment	4.8	(2.0)	2.6	(1.0)	1.0	(0.0)	2.6	(1.0)	5.0	(2.1)
**Knee**										
Sagittal moment	1.8	(0.8)	1.3	(0.4)	1.0	(0.0)	1.3	(0.4)	1.8	(0.8)
Frontal moment	1.9	(1.0)	1.3	(0.5)	1.0	(0.0)	1.3	(0.6)	2.0	(1.1)
Horizontal moment	1.6	(0.5)	1.2	(0.3)	1.0	(0.0)	1.3	(0.4)	1.9	(0.8)
**Ankle**										
Sagittal moment	1.2	(0.3)	1.1	(0.2)	1.0	(0.0)	1.1	(0.3)	1.4	(0.5)
Frontal moment	2.3	(1.5)	1.5	(0.8)	1.0	(0.0)	1.4	(0.8)	2.3	(1.4)
Horizontal moment	1.5	(0.6)	1.1	(0.3)	1.0	(0.0)	1.2	(0.4)	1.5	(0.7)

Note: NRMSE error indicates the ratio of the corresponding NRMSE to that in the ZERO condition.

## Data Availability

The datasets used and/or analyzed during the current study are available from the corresponding author on reasonable request.

## Supplementary Materials

The following supporting information can be downloaded at: https://www.mdpi.com/article/10.3390/s24165096/s1, Table S1 Results of multiple regression analysis. Figure S1. PCLs of PCV7 from the acceleration matrix. Table S2. Results of correlation coefficients between PCS and walking speed.
