# Supplementary material for: Errors in Estimating Lower-Limb Joint Angles and Moments during Walking Based on Pelvic Accelerations: Influence of Virtual Inertial Measurement Unit’s Frontal Plane Misalignment
Source: Sensors (Basel). 2024 Aug 6;24(16):5096. doi: 10.3390/s24165096 (PMC11359074; doi:10.3390/s24165096)
Supplement: Supplementary file 1 [file sensors-24-05096-s001.zip › sensors-3112406-supplementary.pdf]

Supplementary Material

Additional analysis S1

Methods

To investigate the mechanism behind the significant impact of IMU misalignment on estimated pelvis and hip angles in the frontal plane, a PCA was conducted on the joint angle matrix (including only the pelvis and hip angles in the frontal plane) and the acceleration matrix. Subsequently, a multiple regression analysis was performed, using all PCSs from the acceleration matrix as independent variables and PCS1 from the joint angle matrix (including only the pelvis and hip angles in the frontal plane) as the dependent variable. Finally, the PCL of the PCV with the highest t-value was observed.

Results

PCV7 from the acceleration matrix and PCV1 from the joint angle matrix showed a strong relationship (Table S1, where PCS1 from the joint angle matrix is the dependent variable). As shown in Figure S1, PCV7 reflects the acceleration along the x-axis.

Table S1. Results of multiple regression analysis.

	β	t value	p value
(Intercept)	0.00	0.00	1.000

PCV1	0.14	5.36	**0.000**
PCV2	−0.04	−1.70	0.090
PCV3	0.13	5.16	**0.000**
PCV4	−0.31	−12.07	**0.000**
PCV5	0.05	1.93	0.054
PCV6	0.27	10.52	**0.000**
PCV7	−0.74	−28.65	**0.000**
PCV8	0.11	4.40	**0.000**
PCV9	−0.20	−7.78	**0.000**
PCV10	−0.02	−0.59	0.558
PCV11	−0.03	−1.11	0.270
PCV12	0.05	2.07	**0.039**
PCV13	−0.06	−2.40	**0.017**
PCV14	−0.07	−2.87	**0.004**
PCV15	−0.03	−1.21	0.227
PCV16	0.01	0.27	0.785
PCV17	0.05	1.92	0.055
PCV18	−0.07	−2.71	**0.007**
PCV19	−0.08	−3.12	**0.002**
PCV20	0.00	0.06	0.955
PCV21	0.02	0.88	0.381

β: Standard partial regression coefficient

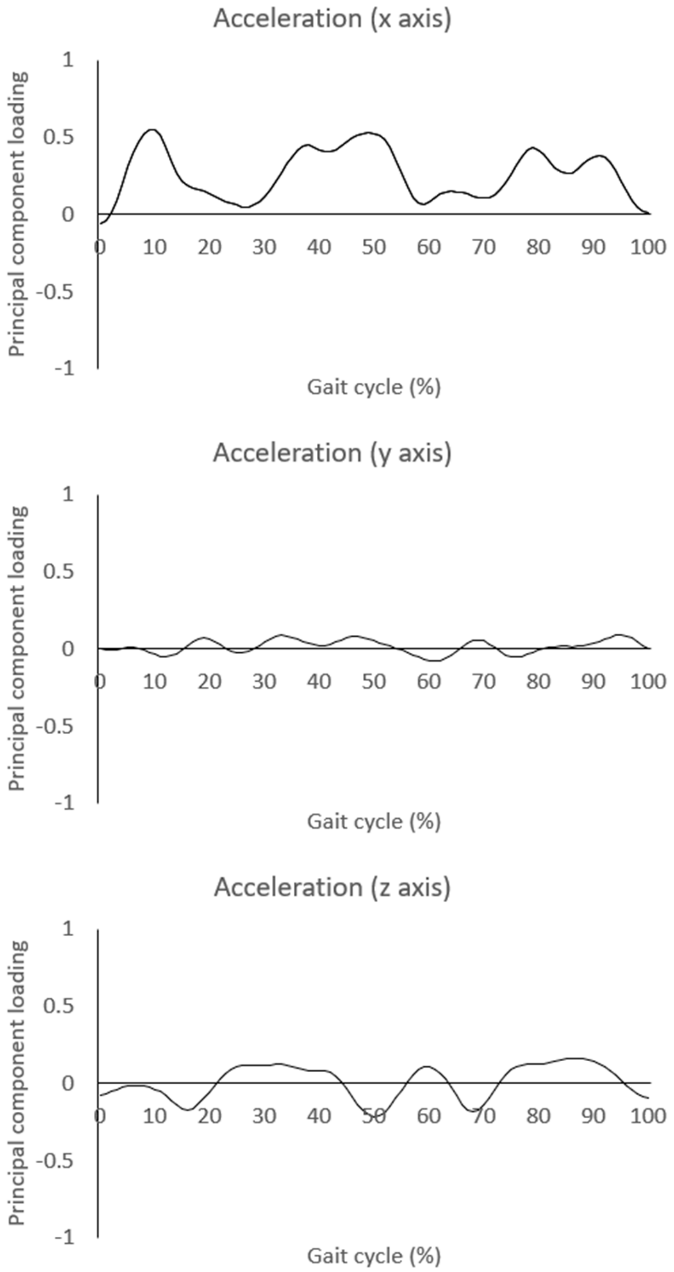

Figure S1. PCLs of PCV7 from the acceleration matrix

Additional analysis S2

Methods

We performed a PCA on the acceleration matrix and subsequently calculated the

correlation coefficients between walking speed and each PCV.

Results

Strong relationships were observed between walking speed and certain PCVs, such as PCV5, PCV7, and PCV9.

Table S2. Results of correlation coefficients between PCS and walking speed.

	**r**
PCV1	0.03
PCV2	0.00
PCV3	0.07
PCV4	0.54
PCV5	0.80
PCV6	0.03
PCV7	0.85
PCV8	0.08
PCV9	0.82
PCV10	0.27
PCV11	0.17
PCV12	0.54
PCV13	0.19
PCV14	0.49
PCV15	0.00
PCV16	0.67
PCV17	0.11
PCV18	0.50
PCV19	0.39
PCV20	0.60
PCV21	0.42
